# Plasma degradation of contaminated PPE: an energy-efficient method to treat contaminated plastic waste

**DOI:** 10.1038/s41529-023-00350-9

**Published:** 2023-04-19

**Authors:** Mariano Marco Tobías, Michelle Åhlén, Ocean Cheung, David G. Bucknall, Martin R. S. McCoustra, Humphrey H. P. Yiu

**Affiliations:** 1 Chemical Engineering, School of Engineering and Physical Sciences, Heriot-Watt University, Edinburgh, EH14 4AS UK; 2 Nanotechnology and Functional Materials, Department of Materials Science and Engineering, Uppsala University, Ångströmlaboratoriet, Lägerhyddsvägen 1, 752 37 Uppsala, Sweden; 3 Institute of Chemical Sciences, School of Engineering and Physical Sciences, Heriot-Watt University, Edinburgh, EH14 4AS UK

**Keywords:** Materials chemistry, Polymer chemistry

## Abstract

The use of PPE has drastically increased because of the SARS-CoV-2 (COVID-19) pandemic as disposable surgical face masks made from non-biodegradable polypropylene (PP) polymers have generated a significant amount of waste. In this work, a low-power plasma method has been used to degrade surgical masks. Several analytical techniques (gravimetric analysis, scanning electron microscopy (SEM), attenuated total reflection-infra-red spectroscopy (ATR-IR), x-ray photoelectron spectroscopy (XPS), thermogravimetric analysis/differential scanning calorimetry (TGA/DSC) and wide-angle x-ray scattering (WAXS)) were used to evaluate the effects of plasma irradiation on mask samples. After 4 h of irradiation, an overall mass loss of 63 ± 8%, through oxidation followed by fragmentation, was observed on the non-woven 3-ply surgical mask, which is 20 times faster than degrading a bulk PP sample. Individual components of the mask also showed different degradation rates. Air plasma clearly represents an energy-efficient tool for treating contaminated PPE in an environmentally friendly approach.

## Introduction

Treatment of contaminated medical personal protective equipment (PPE) wastes (such as plastic surgical masks and gloves) is problematic due to the hazards associated with handling potentially infectious materials. This problem has been dramatically magnified by the SARS-CoV-2 (COVID-19) pandemic. According to the World Health Organization (WHO), by March 2020, it was estimated that healthcare professionals alone were using ~89 million masks every month^1^. The effect of this surge in the use of PPE and their subsequent disposal has been felt by every part of the world. It was estimated that 3.4 billion single-use facemasks or face shields were discarded daily in 2020^2^. In the city of Jakarta in Indonesia, for instance, some 12,740 tons of medical waste were produced in the first 60 days of the pandemic^3^. Taking face masks as an example, in China, the use of single-use face masks has increased 12-fold since the pandemic began^4^. Another example is Catalonia where medical waste including overalls, face masks and gloves increased from 275 tons in March 2020 to 1200 tons in April 2020^5^. The problem is aggravated since the coronavirus can survive on surfaces (e.g., metal, glass and plastic) for up to 9 days^6^. Moreover, since many items of PPE are made from plastics, which take decades to degrade in the natural environment^7^, mismanagement of PPE waste presents a significant ecological challenge. Therefore, effective but safe, energy-efficient and sustainable treatments for such a large amount of waste material must be considered to mitigate further hazards to both health and also the environment.

**Fig. 1:**
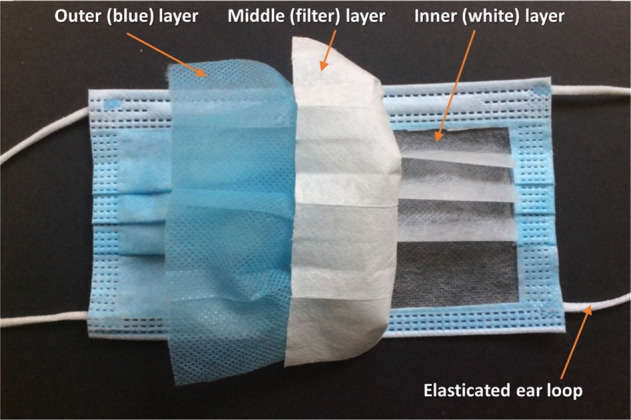
An image of a surgical mask. The three layers of a typical surgical mask are shown.

Amongst commonly used PPE, disposable 3-ply surgical masks (see Fig. 1) have proven to be most popular as they are relatively effective, accessible and provide a cost-effective solution against virus transmission^8^. These 3-ply surgical masks are typically manufactured from polypropylene (PP), although other plastics including polystyrene (PS), polycarbonate (PC), polyethylene (PE) and polyester (PES) have been utilised^9^. Most of the PPE waste from healthcare establishments (hospitals, clinics and care homes) requires decontamination prior to disposal in landfill unless being incinerated in designated facilities, as is common practice in North America, the EU and the UK^10^. However, incineration presents several environmental challenges. Firstly, the incineration of plastics produces air pollution control (APC) residues, i.e. solid by-products. APC residues are hazardous waste and further treatment is necessary for its disposal^11^. Secondly, and more importantly, the flue gases produced during incineration also need to be treated as they may contain large quantities of pollutants, notably toxic organic compounds including dioxins and furans^12^. Furthermore, the costs associated with medical waste disposal are considerable, with pre-pandemic medical waste disposal costs of around £450 per tonne in the UK and $790 per tonne in the United States^10^. Transportation of these biohazard materials from healthcare establishments to incineration sites adds another challenging layer to the problem^13^. Furthermore, the current generation of incineration systems is not designed to recover any of the organic combustion by-products as potential starting materials for polymerisation (i.e., chemical recycling). Consequently, the cradle-to-grave linear usage and incineration of PPE do not match any goals for materials circularity (i.e., reuse or recycling) and therefore ultimately adds significantly to the global greenhouse gas and environmental burden.

An alternative to direct incineration after use is the sterilisation of contaminated PPE waste on-site prior to disposal to a landfill. From a circular economy viewpoint, sterilisation may also allow the reuse and closed-loop recycling of PPE without the loss of plastic out of the materials system that would occur through the use of incineration^14^. An ideal solution would be an optimal balance between sterilising the PPE waste to remove any biohazard and preserving its structural integrity and thereby allowing reuse in their primary purpose of protecting users. A circular economy approach in the management of PPE waste can be achieved if such a balance is found.

Plasma technologies have been exploited for a wide range of applications including plastics degradation, surface etching, chemical modification^15^ and even sterilisation. With respect to sterilisation, plasma treatment has also been demonstrated to effectively decontaminate material surfaces by inactivating several key pathogens such as ESKAPE (A group of six nosocomial pathogens: *Enterococcus faecium*, *Staphylococcus aureus*, *Klebsiella pneumoniae*, *Acinetobacter baumannii*, *Pseudomonas aeruginosa*, and *Enterobacter* spp.)^16^, *Escherichia coli*^17,18^, *Enterococcus mundtii*^18^, *Candida albicans*^19^, *Staphylococcus aureus*^17^, multiply-resistant *Staphylococcus aureus* (MRSA)^17,20–22^, and *Staphylococcus epidermidis*^21^), as well as various types of viruses^23–27^. Plasma can even be used for skin disinfection with minimal harmful effects^18,28^. Another advantage of plasma irradiation is that it also produces UV radiation, which is also commonly used for the disinfection of small items^9^, and surfaces^29^ as well as for the treatment of medical wastewater^30^. Furthermore, a test on cold atmospheric pressure (CAP) plasma of antimicrobial resistant (AMR) bacteria showed significant inactivation or reduction in MRSA^31^ with a decrease from 30 × 10^6^ colonies to only ca. 600 colonies after 2–5 s of plasma treatment. With careful control, plasma technologies therefore can also offer a viable, fast, safe and green alternative for the sterilisation and decontamination of PPE wastes.

**Fig. 2:**
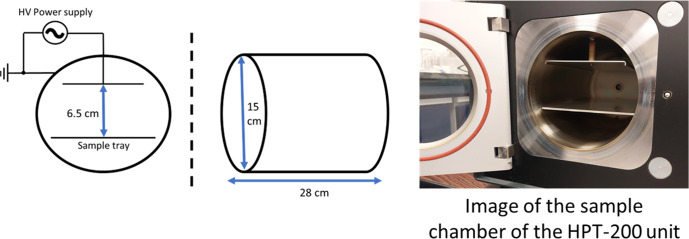
Schematic showing the dimension of the plasma chamber in Henniker HPT-200. Left: the front view of the chamber; middle: the side view of the chamber; right: image of the sample chamber of the HPT-200 unit.

In this study, we have examined the viability of using a cold plasma system (Fig. 2) for the degradation of commercially sourced surgical masks. The weight loss from mask samples was quantified against irradiation time. The changes in the morphology of the PP fibres due to plasma irradiation were monitored using scanning electron microscopy (SEM) while the chemistry of the fibre surface was studied using Fourier transform infra-red spectroscopy (FTIR) and x-ray photoelectron spectroscopy (XPS). The results suggest that cold plasmas offer a viable method for treating contaminated PPE as well as other plastic wastes.

## Results

### Plasma irradiation-induced mass loss

**Fig. 3:**
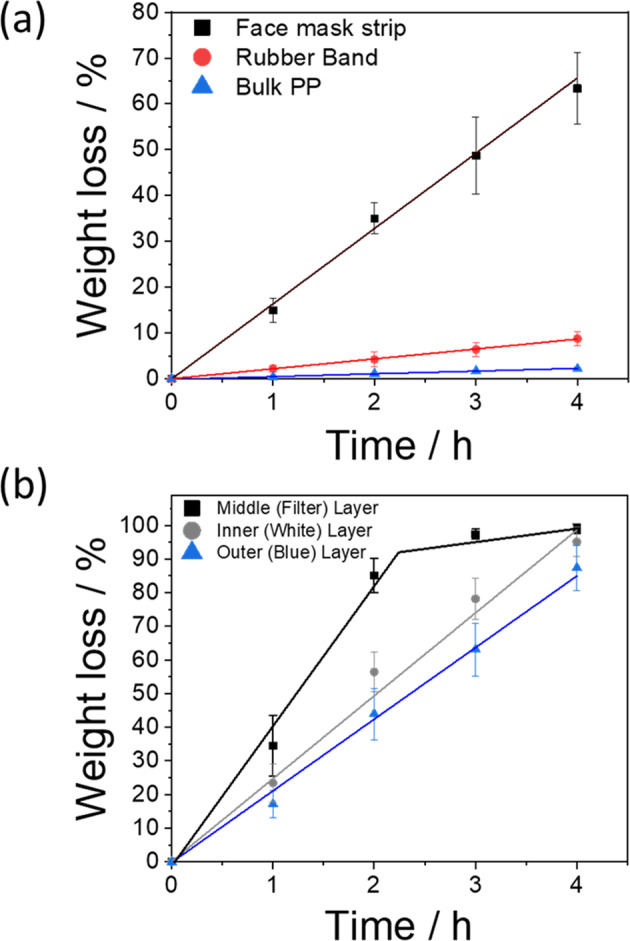
Degradation of masks components. **a** Comparison of the percentage of mass loss for face mask strips (black squares), face mask elasticated ear loops (red circles) and bulk PP against time (blue triangles). The mass loss rates reported in the text are given by the slopes of the individual lines. **b** Comparison of the percentage of mass loss of the three different layers comprising the face mask against time. The error bars represent the standard derivation of the data points.

Mass loss associated with plasma etching of the unfolded mask containing the three layers, as well as the elasticated ear loops, was monitored over a 4 h period and compared against a bulk (solid) PP sheet using a microbalance. As Fig. 3a shows, there is a linear relationship between mass loss and plasma etching time for all irradiated samples. Among these samples, the face mask (all three layers together) was etched at the fastest rate of 0.26 ± 0.03% mass loss per minute of plasma irradiation and a total mass loss of 63.34 ± 7.76% after 4 h. The standard design of these surgical masks has three folds, as seen in Fig. 1. When folded the degradation rate reduces by 0.20 ± 0.02% mass loss per minute giving a total mass loss of 45.50 ± 2.65% after 4 h of irradiation, due to reduced surface area in direct contact with the plasma. In practice, if this technique were to be implemented in healthcare establishments, contaminated masks are unlikely to be stretched before plasma treatment.

Compared with the mask sample, bulk PP exhibited the slowest mass loss rate of 0.01 ± 0.01% per minute. The significant difference in the etching rates of the PP between the face mask and the solid sample is due to the non-woven fibre morphology of the PPE presenting an extremely high surface-area-to-volume (*S*–*V*) ratio to the plasma compared to the solid surface of the bulk PP. Given that low-energy plasma affects the surface of the material, the high *S*–*V* ratio fibre surfaces will etch faster than the bulk sample for a nominally similar macroscopic surface area of the sample. The etching of the elasticated ear loop is seen to be slower than that of the face mask, with a mass loss of 8.75 ± 1.5% loss after 4 h plasma irradiation, i.e., a rate of 0.04 ± 0.01% per minute.

These results are very promising given that low power (200 W) irradiation was used for this experiment resulting in nearly half of the face mask mass being lost after just 4 h. In principle, increasing the power applied to the plasma will accelerate the degradation of the face mask. The relationship between the rate of degradation and the power of the plasma has been demonstrated in the literature^32^. This is also supported by the behaviour of bulk PP under plasma irradiation as shown in Supplementary Fig. 1. The rate of weight loss for a range of power levels between 60 and 200 W exhibits what appears to be exponential behaviour.

When the three individual layers of the face mask were irradiated separately, different mass loss rates were also observed, as depicted in Fig. 3b. Among these layers, plasma irradiation induced the strongest etching effect on the middle (filter) layer with a loss of 99.0 ± 1.5% after 4 h irradiation, while the blue (outer) layer was the least affected with a weight loss of 87.4 ± 6.7%. The degradation rates for the individual layers are higher than for the 3-ply samples. In the latter case, the plasma interacts predominantly with the outer (blue) layer, which also screens the plasma interactions with the underlying two layers significantly reducing degradation happening as efficiently as in separated layers where they are completely exposed. Although the three layers of the face mask are all PP, they were manufactured from different methods. The middle filter layer is commonly manufactured by melt-blown extrusion while the other two (outer and inner) layers are made using the spun-bond technique. Our observations suggested that the manufacturing method could also influence the behaviour of materials under plasma irradiation.

### Morphological changes induced by plasma treatment

**Fig. 4:**
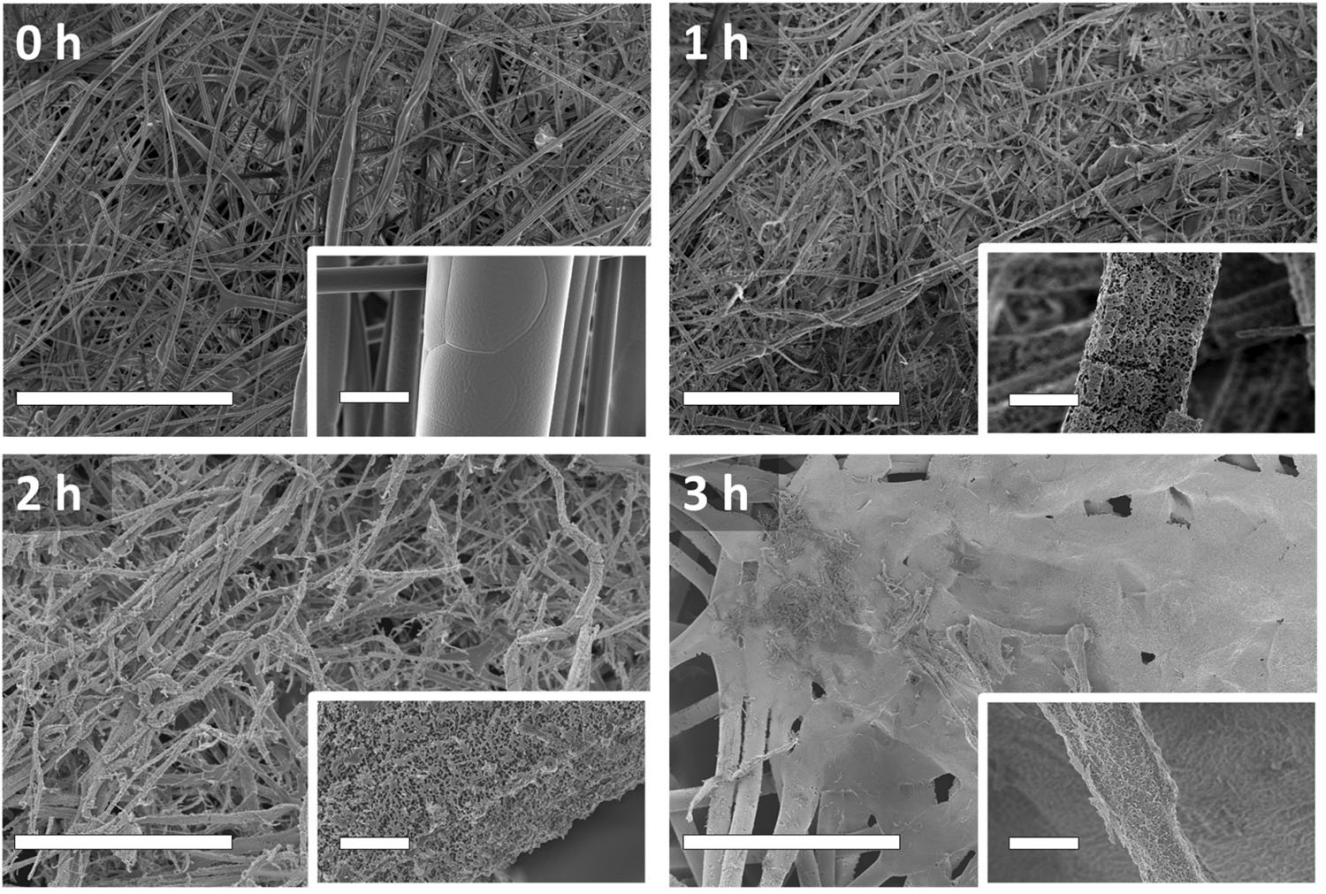
SEM images of the middle (filter) layer of a mask. SEM images of fibres in a face mask record at the noted magnifications after plasma treatment for 0–4 h of the middle (filter) layer. Scale bar = 200 μm (6 μm in insets).

The effect of plasma treatment on the morphology of the PP microfibers in each layer of the mask was analysed using SEM. Figure 4 shows a comparison of the morphology of the middle (filter) layer, which showed the highest degradation rate, after irradiation with air plasma for 1, 2, and 4 h with a comparison to an un-irradiated pristine sample. From the low-magnification images, changes in the fibre morphology can be seen to occur between 1 and 2 h after plasma irradiation, leading initially to a reduction in average fibre length. After 4 h of irradiation, significant changes in morphology are observed with regions showing localised melting. High-magnification images (figure insets) show additional changes to the fibre surface with increasing degree of etching causing pitting after as little as 0.5 h plasma irradiation Supplementary Figure 2. The surface etching also coincides with an increase in the average width of the fibres (Supplementary Fig. 3) due to the loss of the thinner fibres, in addition to the fusion of thinner fibres into thicker fibres.

Although the three layers were all etched under plasma irradiation, the etching of the outer (blue) layer and the inner (white) layer is less pronounced than that of the middle (filter) layer (Supplementary Fig. 4). The surface of the fibres in the middle (filter) layer was etched to generate pores, with an increase in the degree of etching up to 1.5 h of plasma irradiation (Supplementary Fig. 2). After 2 h, onset of fibre melting and fusion was also observed. After 4 h, the porosity due to etching was significantly reduced, caused by melting and fusion of the fibres after prolonged plasma treatment, as shown in the SEM images in Supplementary Fig. 5.

### Surface characterisation of plasma-irradiated materials by ATR-IR

**Fig. 5:**
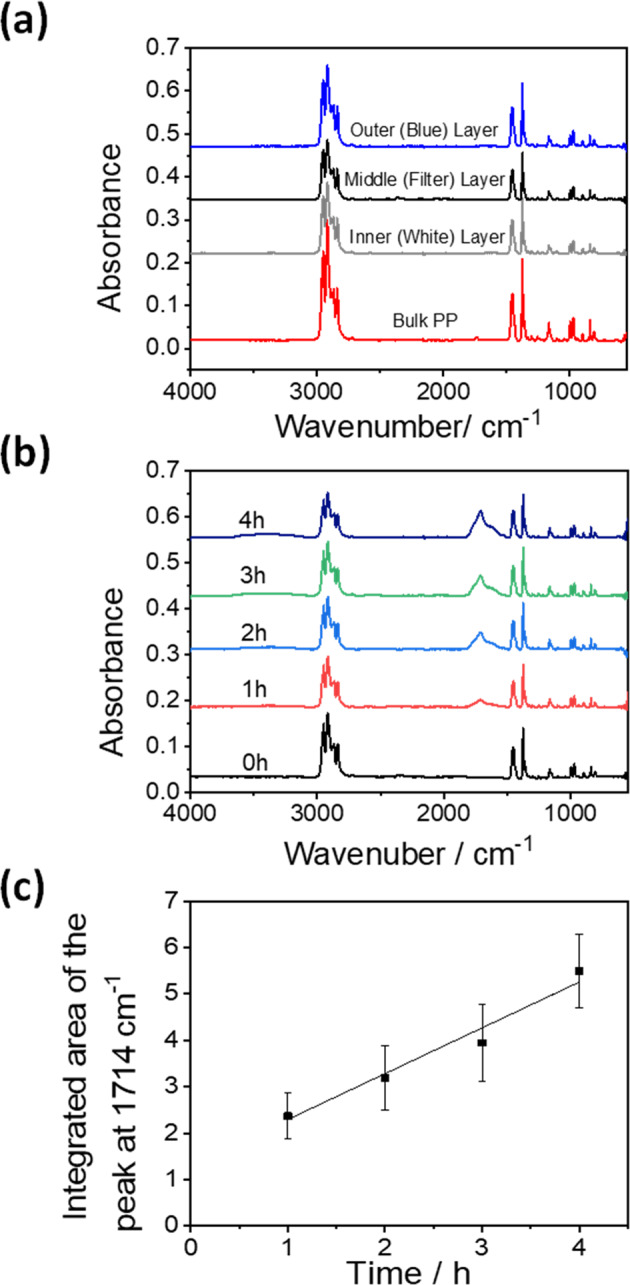
FTIR analysis of mask degradation. **a** Typical ATR-IR spectra recorded for each layer of the three-ply face mask in comparison to that of standard bulk PP sample prior to plasma irradiation. This clearly identifies the mask material as PP. **b** ATR-IR spectra of a face mask sample as a function of irradiation time with 200 W of plasma. Note the appearance of the broad features centred around 1714 cm^−1^ as the face mask material is etched with the plasma. **c** Integrated absorbance of the features at 1714 cm^−1^ from the ATR-IR spectra of a face mask sample as a function of plasma irradiation time. The error bars represent the standard derivation of the data points.

To monitor the changes in the surface chemistry of the samples caused by plasma irradiation, ATR-IR spectroscopic measurements were performed. Figure 5a shows the comparison of the IR spectra of bulk PP with the mask samples. The most notable plasma-induced surface changes on the FTIR spectra of the 3-ply strip samples are shown in Fig. 5b. A peak with a shoulder at around 1714 cm^−1^, which can be assigned to C=O stretching of carboxyl groups, clearly grew in intensity with increasing plasma exposure time. Another broad peak at 3300 cm^−1^, assigned as O–H stretching of hydroxyl groups, does likewise. A measure of the amount of the carbonyl moiety can be determined from the integrated areas of the 1714 cm^−1^ peak when plotted as a function of irradiation time (Fig. 5c). The amount of the carbonyl feature develops rapidly within the first hour of irradiation after which the growth rate slows, possibly indicating saturation of the surface at longer times.

The ATR-IR spectrum of the plasma irradiated bulk PP is compared to that of the 3-ply material PP mask material in Supplementary Fig. 6. The bulk PP sample post-irradiation shows broad features appearing at around 1500–1800 and 3200–3600 cm^−1^; corresponding to those observed in the 3-ply PP mask material. However, the bulk PP bands appear to be shifted to somewhat lower wavenumbers. This is likely a consequence of the detailed nature of the PP material employed in the mask. Clearly, the three PP layers from the face mask behave in a very similar fashion to that of bulk PP (Supplementary Fig. 6). The intensity of the C–H stretching band decreased with plasma irradiation time; while the appearance of O–H stretching and C=O stretching bands were noted. In contrast, the FTIR spectra for the elasticated ear loops during irradiation show little observable change (Supplementary Fig. 7), with no new peaks appearing during irradiation. This is consistent with the weight loss results in Fig. 2a, where only 8.75% weight loss was recorded. The growth of IR peaks for C=O and O–H groups in the spectra for the mask sample suggests that the plasma treatment triggered the formation of ketone and carboxylic acid groups as a result of the incorporation of oxygen atoms from the air plasma during the etching of the PP layer surfaces. This is a key observation as it suggests that surface modification is a pre-requisite to etching and that the generation of simple oxygenated hydrocarbon species in the gaseous stream may offer an opportunity for the production of useful aldehydes, ketones and carboxylic acids.

### Surface characterisation of plasma irradiated materials by XPS

**Fig. 6:**
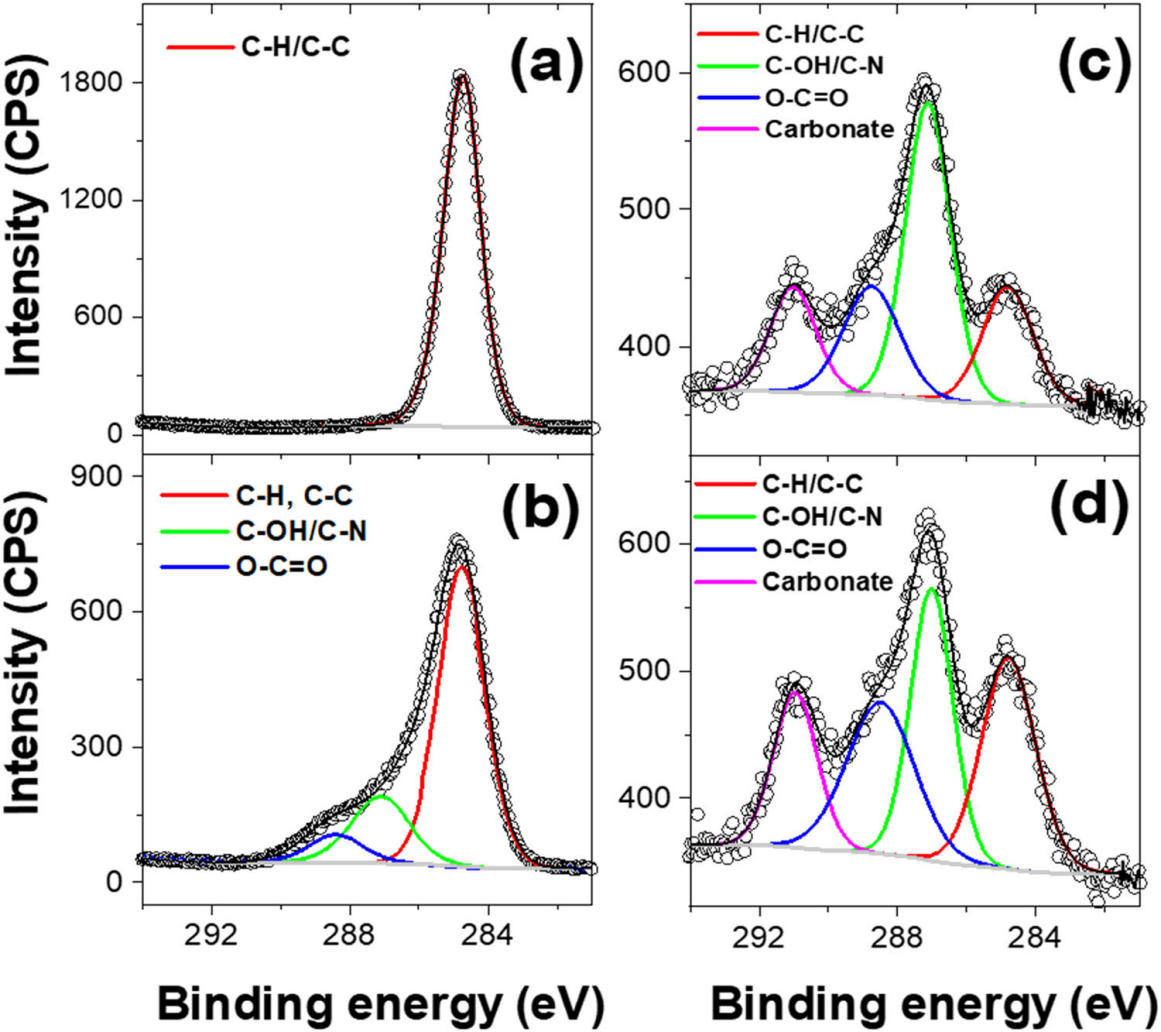
XPS analysis of the plasma-treated middle (filter) layer. XPS data from un-irradiated (**a**) and irradiated samples after **b** 1 h, **c** 2 h and **d** 3 h. Experimental data (circles) with solid lines from fit data as indicated. Assignments of surface chemical moieties based on observed chemical shifts are given in the figures.

To further understand the changes in surface chemistry induced by the plasma treatment, XPS was also employed, as its combination with IR is well-established in the literature as a means of identifying chemical changes in the surface of polymers^33^. The combination of IR group frequencies with XPS chemical shift data obtained from high-resolution XPS allows for the definitive identification of polymer surface-bound species. The high-resolution 1*s* core level XPS data for C, N and O obtained on our samples is summarised in Fig. 6. The XPS results confirm the substantial modification of the surface chemistry in the plasma-treated PP mask samples initially observed in the infra-red spectra.

Fitting of the high-resolution XPS data suggests the presence of multiple chemically shifted components consistent with the addition of O atoms to the hydrocarbon chains. The presence of “oxidised carbon species” at the surface can be seen to emerge after 0.5 h of treatment in both the C and O 1*s* data (see Supplementary Fig. 8). The appearance of additional components in the C 1*s* data at around 286.6 and 288.7 eV, and in the O 1*s* data at around 533.7 and 532.8 eV, is consistent with the presence of C–OH and O–C = O groups, respectively^34^. This confirms the preliminary observations from the IR measurements and indicates the presence of surface carboxyl and hydroxyl groups. This is not inconsistent with the known chemistry in air plasma which is dominated by O atom (both ^3^P and ^1^D), and unless scrupulously dried, OH radical chemistry. H atom abstraction by O (^3^P) and OH is the likely initiator of radical chemistry at the polypropylene surface with subsequent chemistry not dissimilar to low-temperature hydrocarbon oxidation in the gas and liquid phase^35–37^. The reactions are likely to favour tertiary sites first, followed by secondary and finally primary sites^38^. While O (^1^D) insertion in C–H bonds and H_2_ elimination provides an additional route to C=O moieties in the polymer surface^39,40^.

The emergence of surface-bound N species is clearly observed in the N 1*s* XPS data with the appearance of features at around 401.3 and 400.9 eV that are consistent with N–(C = O)– and C-NR_2_ (R = C, H) moieties. This is not surprising given the presence of N atoms in the air plasma^35,36,41,42^. However, ground state N (^4^S) is known to be unreactive to saturated hydrocarbon species and is only likely to react with CH_2_ or CH radical centres already present on the polymer surface yielding chemically bound N moieties^43,44^. Electronically excited N (^2^D) atoms have a richer chemistry in being able to react with saturated hydrocarbons yielding >C=NH functionality that is open to further chemistry^45–47^. The growth of a C 1*s* peak at around 290 eV suggests the presence of a metal carbonate, and the presence of N 1*s* peaks at around 397 and 404–405 eV might be indicative of metal nitrides and nitrates. A potential source of this metal may be from the residual catalyst used in the PP polymerisation production processes.

### TGA/DSC and wide-angle X-ray scattering (WAXS)

**Fig. 7:**
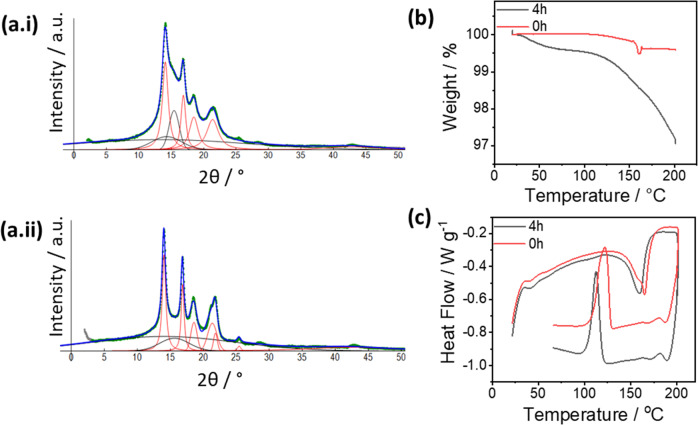
WAXS, TGA and DSC analyses for the filter layer. **a** WAXS plots for the middle filter layer (**a.i**) before and (**a.ii**) after plasma irradiation for 4 h. Comparison on **b** weight loss curves from the TGA and **c** heat flow from DSC measurement of the middle filter layer before and after plasma irradiation. Results from other layers can be found in SI.

To study changes in the degree of crystallinity of the PP both TGA/DSC and WAXS measurements were performed. Regarding the degree of crystallinity, two methods were employed. The values of the crystallinity from the two techniques are tabulated in Supplementary Table 1. For both methods, it was observed that there is a decrease in the degree of crystallinity in all the layers after plasma irradiation. For WAXS measurements, the diffraction pattern of the three individual layers before and after plasma irradiation is shown in Fig. 7a and Supplementary Fig. 9. From the TGA measurements (Fig. 7b and Supplementary Fig. 10), it can also be observed that for each individual layer the amount of weight loss increases after being irradiated. When compared between the three layers, the middle filter layer again showed an increase in weight loss between 100 and 200 °C, with an increase of over 3% after irradiation. This indicated the polymers have been degraded to smaller and more volatile organics. The DSC measurements (Fig. 7c and Supplementary Fig. 11) show that the melting temperatures (*T*_m_) of the individual layers are also decreased after plasma irradiation. This observation could also be linked to the fragmentation of large polymer molecules. Once again, the middle filter layer showed the largest decrease in *T*_m_ because the highest polymer degradation was observed due to plasma irradiation. All these results support the hypothesis that the polymer chains degrade into smaller fragments due to plasma irradiation. Moreover, during degradation, oxygen functional groups (aldehydes, ketones, and carboxylic acids) are introduced as proved by FTIR and XPS measurements. The introduction of these groups could also hinder the stacking of the polymer chains thus reducing the degree of crystallinity and lowering the melting temperature.

## Discussion

Plasma treatment allows rapid degradation of PP microfibres in non-woven textiles, for the middle layer of PPE masks, where 90% weight loss was recorded after 4 h of treatment. The degradation processes between the different layers were shown to vary considerably. When a 3-ply mask sample was treated, the middle filter layer was seen to etch significantly generating highly porous fibres, whilst the outer blue layer did not show significant changes in morphology. This retention of the structural integrity of the outer blue layer is remarkable since all these layers were made from PP and are therefore chemically the same. The difference in the etching of the different mask filter layers suggests that the response to plasma irradiation is influenced by the processing method of the polymers. The middle layer is usually manufactured using the “non-woven melt-blown process” while the outer layers were made from the “non-woven spun-bond process”. In the spun-bond processing, drawing generates fibres not only with a relatively narrow fibre diameter distribution but also a high degree of chain orientation, leading to mechanically strong fibres. By contrast, typically melt-blown processing uses lower molecular weight polymers in order to reduce the melt viscosity, but the high-temperature airflow used attenuates the melt stream creating fibres but with a variation of diameters both along and between fibres. Both factors contribute to fibres that not only have poorer mechanical properties compared to spun-bond fibres but are also less resistant to plasma treatments^48^. Indeed, other minor components such as dyes and plasticisers can also affect the rate of weight loss. Since there is a lack of information regarding the identity and the amount of these minor components, their effect can be difficult to identify and quantify.

However, the instrument used in our study was designed for low-power (a maximum of 200 W) cleaning, etching and deposition of polymers, not for large-scale plasma degradation. Although the initial results were impressive, it is difficult to estimate how much waste mask material it could handle. For future development in this direction, a customised reactor is needed solely for degradation studies on a larger scale. Furthermore, the power used to generate the plasma needs to be significantly increased to allow faster degradation rates. In respect of the power consumption, we can compare that of a muffle furnace (up to 1000 °C) of a similar internal volume (16 × 16 × 17 cm), typically used for pyrolysis, having a power consumption of ca. 4000 W, with the maximum 200 W applied to the plasma in the work described. This is a 20-fold increase in power input. Although these figures may not be directly comparable, they do indicate that cold plasma processing generally consumes much less energy and hence generates much less CO_2_.

**Fig. 8:**
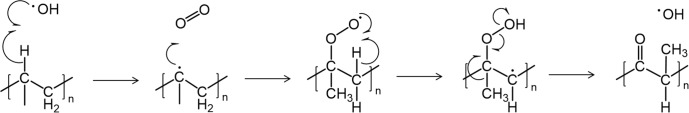
Proposed cold-flame oxidation mechanism. Proposed mechanism for the cool-flame oxidation for a polypropylene framework to form a carbonyl group at the initial stage.

Regarding the degradation mechanism, we may expect a mechanism like that of classical cool-flame oxidation of short-chain hydrocarbons in the gas phase^49,50^. In this mechanism, oxidation is initiated by ·OH free radicals, generated by the cold plasma, which results in C_*n*_H_2*n*+1_· (alkyl) free radicals, as shown in Fig. 8. These unstable alkyl radicals can react readily with O_2_ in the air to form β- and γ-hydroperoxyalkyl radicals. Carbonyl species can then form through intermolecular rearrangement (e.g., Me shift or Et shift). Further oxidation of the carbonyl products will form carboxylic acids. These groups were all identified by FTIR and XPS. Based on this mechanism, as the oxidation progresses, small organic molecules including small carbonyls, carboxylic acids, or even short hydrocarbons could be produced from plasma irradiation of the polypropylene fibres of 3-ply masks. This chemistry will result in a number of gaseous products including short-chain oxygenates (aldehydes, ketones and related species) and carbon monoxide, as well as carbon dioxide and water due to prolong irradiation as in complete combustion. Further work is required to detail these products.

Recently, single-use surgical mask usage has sky-rocketed due to the exponential growth in their use caused by the COVID-19 pandemic with the result that there is a global problem with plastic waste. It is also speculated that surgical masks could be a new source of microplastics^51,52^. It is reasonable to assume that microplastics could be produced if the microfibres (as seen in Fig. 4) degrade in an uncontrolled manner and eventually enter the environment via aquatic routes or food chains^53^. The current methods for treating disposable masks, e.g., landfill and incineration, will not significantly improve this situation^54^. If the masks are to be processed in incineration systems, the mass loss will be close to 100% (except the incombustible component such as the metal wire) due to the high temperature (typically 900–1300 °C). Any morphological changes will be initially due to melting. At such a high temperature, the PP fibres will be expected to undergo fragmentation initially and form short-chain hydrocarbons^55^. Therefore, the surface is unlikely to be oxygenated like those undergone a plasma treatment. As we have shown that cold plasma can degrade masks in a controlled manner, the release of microplastics from disposable masks can therefore be managed systematically.

If recyclable masks are to be developed, the manufacturing processes for the middle layers may need to be changed, possibly adapted to the non-woven spun-bond process. Short plasma treatments (e.g., 10 min) have been used for sterilisation^56^, and this process can be used to sterilise masks after use if the integrity of the PP layers is preserved. This can be implemented in the healthcare sector worldwide, including medical clinics, dental clinics, care homes, and hospitals. Such multi-use surgical masks could significantly reduce plastic waste but also maintain the health and safety of health workers as well as patients. Although this surge of waste masks was triggered by the COVID-19 pandemic, the use of masks in public may remain high for years to come as illustrated by their continued use since the 2003 SARS epidemic in China and South-East Asia. Consequently, plastic waste generated from single-use surgical masks will likely remain high for the next decade.

The benefits of plasma treatment are two-fold. This technique could be exploited as a sterilisation regime for the re-use of masks. Long-time treatments (e.g., up to 4 h) and/or at greater plasma-generating powers can be used for the decomposition of masks, reducing solid waste. It could also potentially be a better alternative to incineration, which is widely utilised in many hospitals/healthcare establishments worldwide, with lower energy requirements and lower emissions of toxic organics. Moreover, incineration also results in the permanent loss of potential added-value materials to CO_2_ emission whilst plasma technologies allow alternative routes for the generation of useful organics such as carbonyl, carboxylic acids, or short hydrocarbon fragments, as suggested from cool-flame oxidation on the polypropylene fibres on masks. These gaseous products are considerably less toxic when compared to those generated from conventional incineration or pyrolysis (e.g., dioxans, PAHs). With reduced power consumption, these useful organics generated from PPE wastes can be another attractive attribute of this technology. Furthermore, when compared to the current PPE recycling method in some hospitals, e.g., thermal compaction, the use of plasma technology does not require materials separation pre-recycling, which is needed in many processes to separate non-PP components from masks (such as elastic bands, metal wire etc.). Such separation processes will highly reduce the efficiency of recycling and increase the cost.

In this work, the degradation of surgical masks via air plasma irradiation was studied. To examine the effects of plasma irradiation, gravimetric analysis, ATR-IR, SEM, XPS, TGA/DSC and WAXS methods were performed. The results show that a 3-ply PP face mask was not only degraded, losing 63.34% of its original weight with a set power of 200 W after 4 h irradiation, but the surface was also modified by the inclusion of C=O and O–H groups potentially allowing the production of value-added chemicals (e.g., aldehydes, ketones, or carboxylic acids) in the etching gaseous stream. Further analysis needs to be done on the gaseous products to confirm the presence of added-value small organics. Additionally, based on the results from the literature, the face masks will be sterilised in less than 1 h of plasma irradiation and could be treated as normal waste instead of as bio-hazardous waste. After 1 h irradiation, the surface of the fibres for the external layer was clearly eroded, while the 2 h irradiated mask strip shows a considerable reduction in its fibre density as well as erosion of their surfaces. Plasma erosion also worked, though less successfully, for the elasticated ear loops with a mass loss of 8.7% after 4 h, compared to 99.0% obtained with the mask filter materials, clearly indicating differences in plasma interactions with polymers of different chemical structures. These results are very promising as the set power used was only 200 W and with a higher set power even faster degradations could be achieved. We also demonstrated that plasma treatments on PPE can be a clean and energy-efficient method to treat these hazardous materials, reducing the environmental burden from landfills. The added value of the proposed degradation route comes from (i) the production of small volatile organics, specifically oxygenates, which are invaluable chemical precursors, and (ii) the potential for integration of the plasma degradation method with catalytic upgrading of the products. Both of these require further exploration of this process to characterise more fully the gas phase production of the degradation chemistry.

## Methods

### Materials and sample preparation

Surgical masks (Fig. 1) were sourced from a commercial outlet (Fisher Scientific). The 3-ply, non-woven fabric was cut into 1.5 ± 0.1 cm by 5.3 ± 0.4 cm strips. The elasticated ear loops were cut into 2.5 cm segments. As a control material, PP sheet (Goodfellow, UK) with a thickness of 1 mm was cut into 1 cm × 1 cm squares with a Proxxon KS 115 table saw. After cutting, the individual PP squares were thoroughly cleaned with distilled water and allowed to air dry.

### Plasma irradiation

Plasma irradiation of all materials was performed using the following protocol. The samples were placed on a metallic tray inside the glow discharge plasma unit (Henniker HPT-200) which had a stainless-steel cylindrical plasma chamber with an aluminium sample tray on which to place samples. The plasma chamber had a diameter of 15 cm and a length of 28 cm for a total volume of 4.95 L (Fig. 2). The plasma unit operates at 40 kHz. A more in-depth description of the plasma unit chamber can be found in the SI. In a typical experiment, a sample was irradiated by a 200 W plasma for time intervals of 30 min up to a total exposure time of 4 h. For samples when evaluating the complete mask (3-ply) strips, the samples were placed with the outer (blue) layer facing the plasma discharge. One sample strip was removed for analysis after each 30-min time treatment interval.

The operation of the automated plasma unit proceeds via three different phases: evacuation, filling with the gas which will support the plasma, and plasma irradiation. The same operating parameters were used in all the plasma treatment experiments. During the evacuation, the plasma chamber was evacuated to ~0.30 mbar. Thereafter, ambient air was introduced into the apparatus at a flow rate of 15 standard cubic centimetres per minute (sccm) until the chamber pressure stabilised at 0.70 mbar. Once this pressure had been reached, the radio frequency (RF) generator was activated, and plasma was produced. Before samples could be extracted after the set plasma treatment time, the unit was vented to the laboratory atmosphere. All plasma treatments for each sample were repeated at least three times to ensure reproducibility.

### Sample characterisations

To avoid contamination when handling the samples, gloves and tweezers were used and the samples were transported between analysis instruments in closed containers. Gravimetric analysis of the weight change of the samples before and after the plasma irradiation was recorded using a five-significant figure microbalance (Avery Berkel). The standard derivation of the results was used to calculate the error. Scanning electron microscopy (SEM) images were collected on a Zeiss Merlin field emission scanning electron microscope (Oberkochen, Germany) operated at an acceleration voltage of 2.5 kV and using an InLens detector. All SEM samples were sputter-coated with a thin layer of Pd/Au prior to imaging. ATR-IR spectra of all the samples were obtained using a Fourier transform infra-red spectrometer (ThermoFisher Nicolet iS5 with an iD5 ATR) in the range of 500–4000 cm^−1^.

Angle-resolved X-ray photoelectron spectroscopy (ARXPS) was performed with a PHI Quantera II scanning XPS microprobe (Chanhassen, USA) using monochromatic Al Kα radiation. All samples were analysed in their pristine states under a constant stream of low-energy electrons to ensure charge neutralisation of the insulating samples. Survey and high-resolution spectra of O 1*s*, N 1*s* and C 1*s* core levels were collected on samples tilted to 90° and using 26 eV pass energy. All spectra were shifted using the C 1*s* peak for adventitious carbon (284.4 eV).

To study the change in the degree of crystallinity both TGA/DSC and wide-angle X-ray scattering (WAXS) measurements were made. In a typical TGA/DSC experiment, a sample (ca. 5 mg) was loaded to a combined TGA/DSC thermobalance (TA Instrument SDT Q600) and heated to 300 °C at a heating rate of 5 °C min^−1^ under a flowing N_2_ atmosphere (75 mL min^−1^); and the DSC melting peak integrated to calculate *Δ*_fus_*H*. This quantity was then divided by the literature value for 100% crystalline PP of 170 ± 3 J g^−1 57^. Wide Angle X-ray scattering (WAXS) was performed with a Panalytical Empyrean diffractometer with a Cu anode source operating at 45 kV and 40 mA. The samples were measured in reflection geometry, with the treated samples mounted on null-reflecting Si substrates. The WAXS scattering data for the untreated and 4 h irradiated samples for each layer, were fitted assuming amorphous (Gaussian) and crystalline (pseudo-Voigt) components fitting the spectra. The percent crystallinity was obtained using the following formula:

1 $$C = \frac{{100.\mathop {\sum }\nolimits_i A_{c,i}}}{{\mathop {\sum }\nolimits_i A_{c,i} + \mathop {\sum }\nolimits_j A_{a,j}}}$$

where *C* is the percentage of crystallinity, and *A*_*a,j*_ and *A*_*c,i*_ are the areas of the individual amorphous (*j*) and crystalline (*i*) peaks.

## Supplementary information

Supplementary Information updated

## Data Availability

The data that support the findings of this study are available from the corresponding author upon reasonable request.
