# Supplementary material for: Plasma degradation of contaminated PPE: an energy-efficient method to treat contaminated plastic waste
Source: Npj Mater Degrad. 2023 Apr 19;7(1):33. doi: 10.1038/s41529-023-00350-9 (PMC10115383; doi:10.1038/s41529-023-00350-9)
Supplement: Supplementary file 1 — Supplementary Information updated [file 41529_2023_350_MOESM1_ESM.pdf]

Electronic Supplementary Information

for

Plasma degradation of contaminated PPE: an energy efficient method to treat contaminated plastic waste

Mariano Marco[a], Michelle Åhlén[b], Ocean Cheung[b], David G. Bucknall[c], Martin R. S. McCoustra[c,*], Humphrey H. P. Yiu[a,*]

[a]Chemical Engineering, School of Engineering and Physical Sciences, Heriot-Watt University, Edinburgh, EH14 4AS, UK

[b]Nanotechnology and Functional Materials, Department of Materials Science and Engineering, Uppsala University, Ångströmlaboratoriet, Lägerhyddsvägen 1, 752 37 Uppsala, Sweden

[c]Institute of Chemical Sciences, School of Engineering and Physical Sciences, Heriot-Watt University, Edinburgh, EH14 4AS, UK

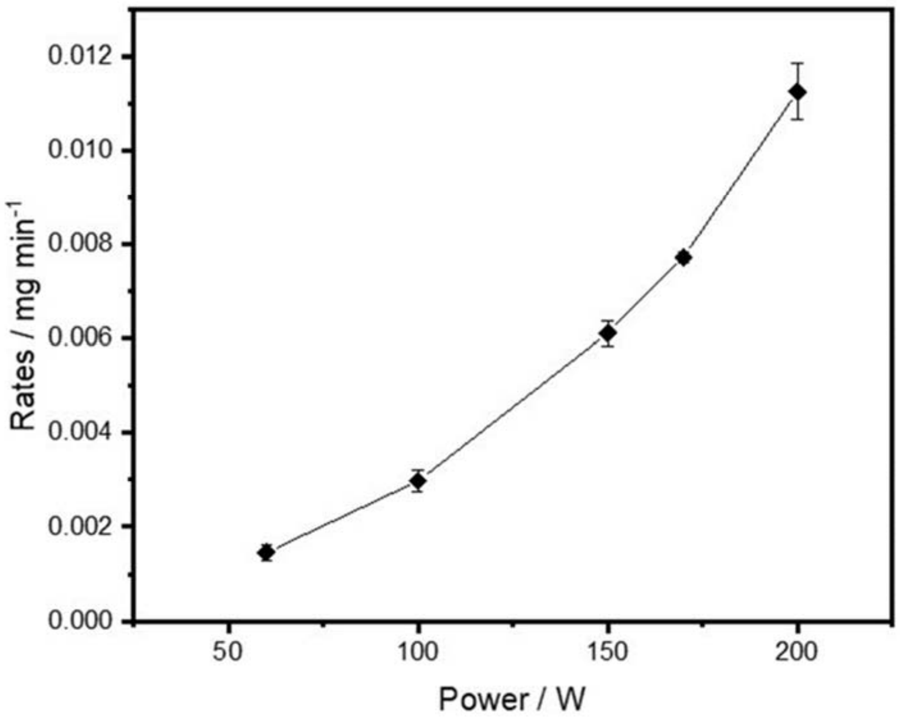

Supplementary Figure 1: Rate of weight loss in mg per minute of plasma irradiation for bulk PP samples for a range of power levels between 60 W and 200 W.

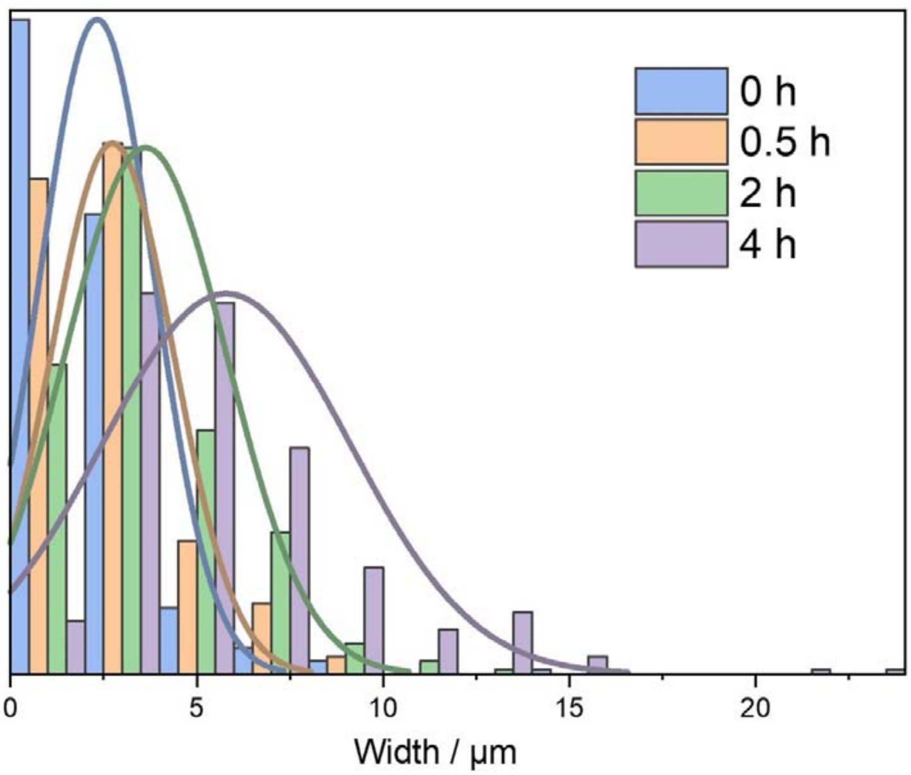

Supplementary Figure 2: Histogram of the distribution fibre widths of the middle (filter) layer following 0, 0.5, 2 and 4 hours of irradiation.

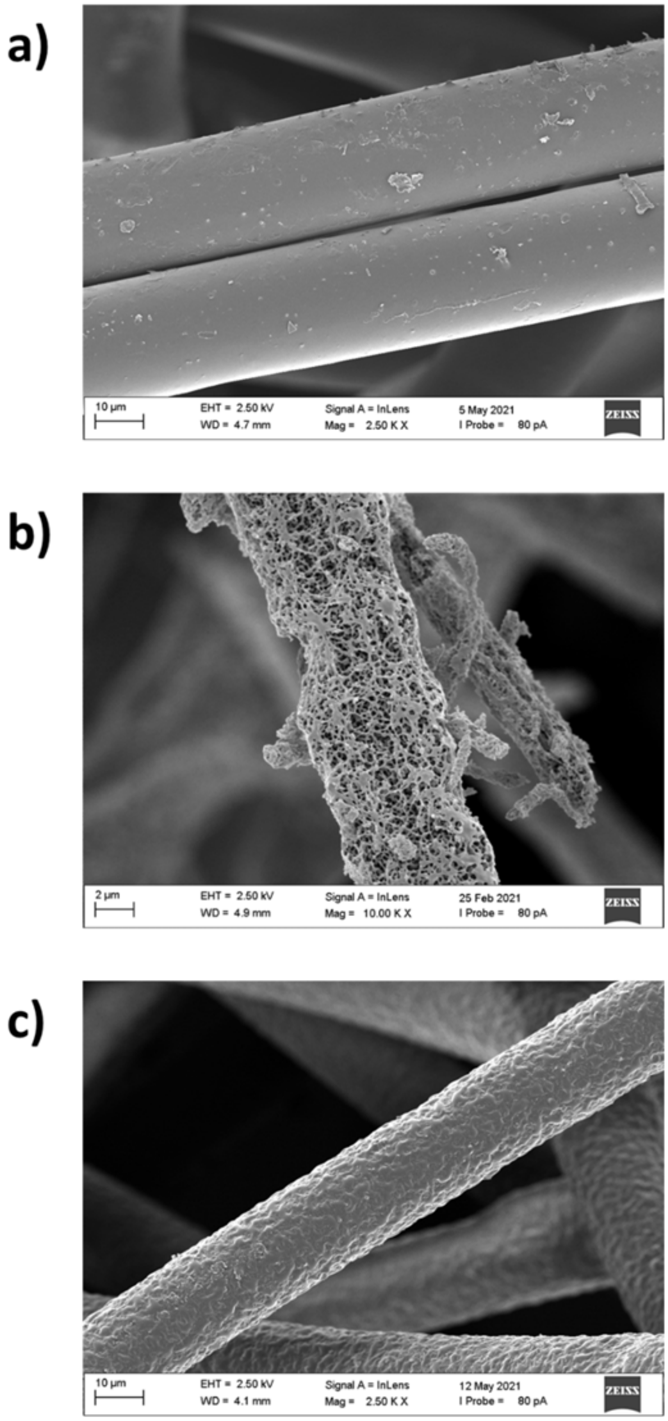

Supplementary Figure 3: SEM images for 2 h irradiated samples of; a) Outer (blue) layer, b) Middle (filter) layer and c) Inner (white) layer.

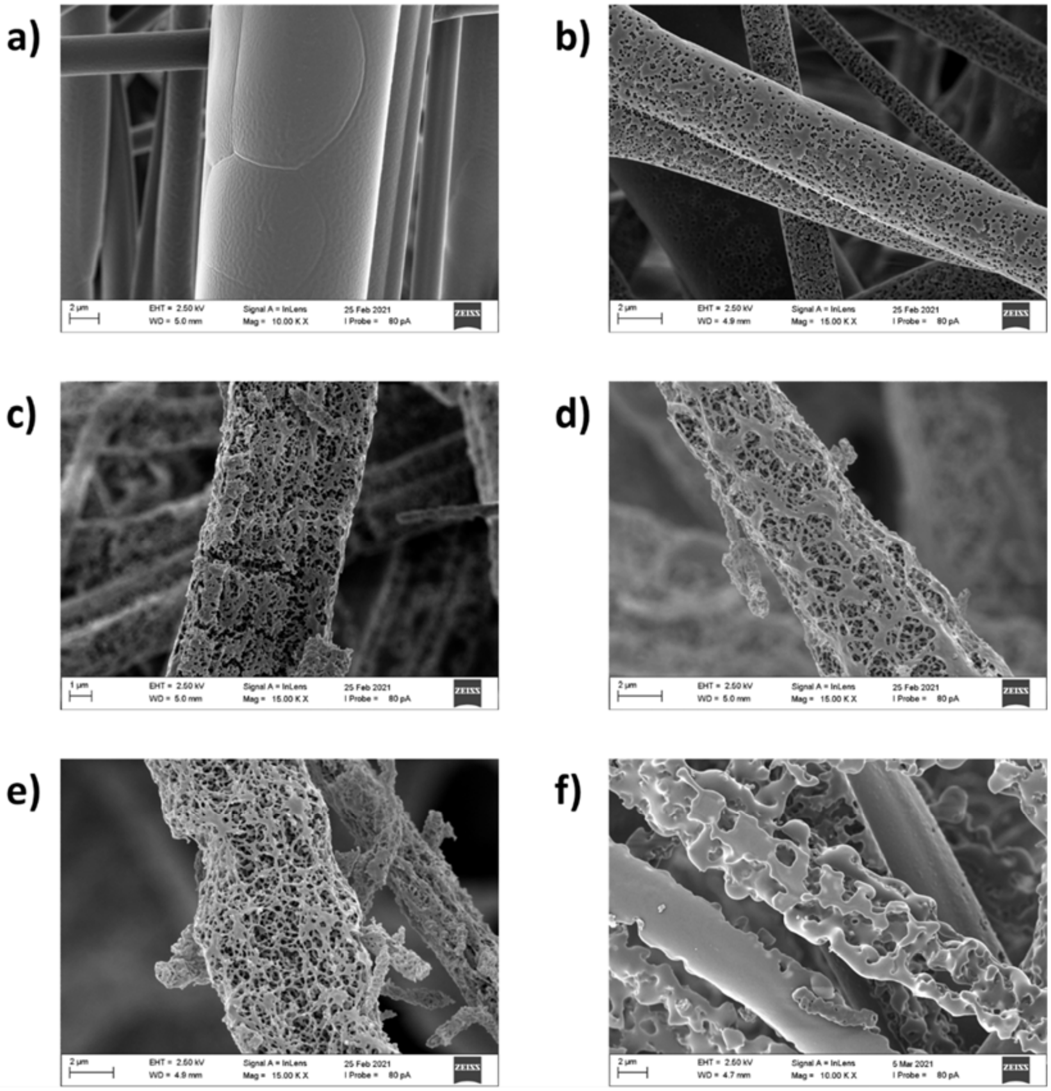

Supplementary Figure 4: SEM images of the Middle (filter) layer for; a) un-irradiated, b) 0.5 h, c) 1 h, d) 1.5 h e) 2 h and f) 3 h irradiated samples.

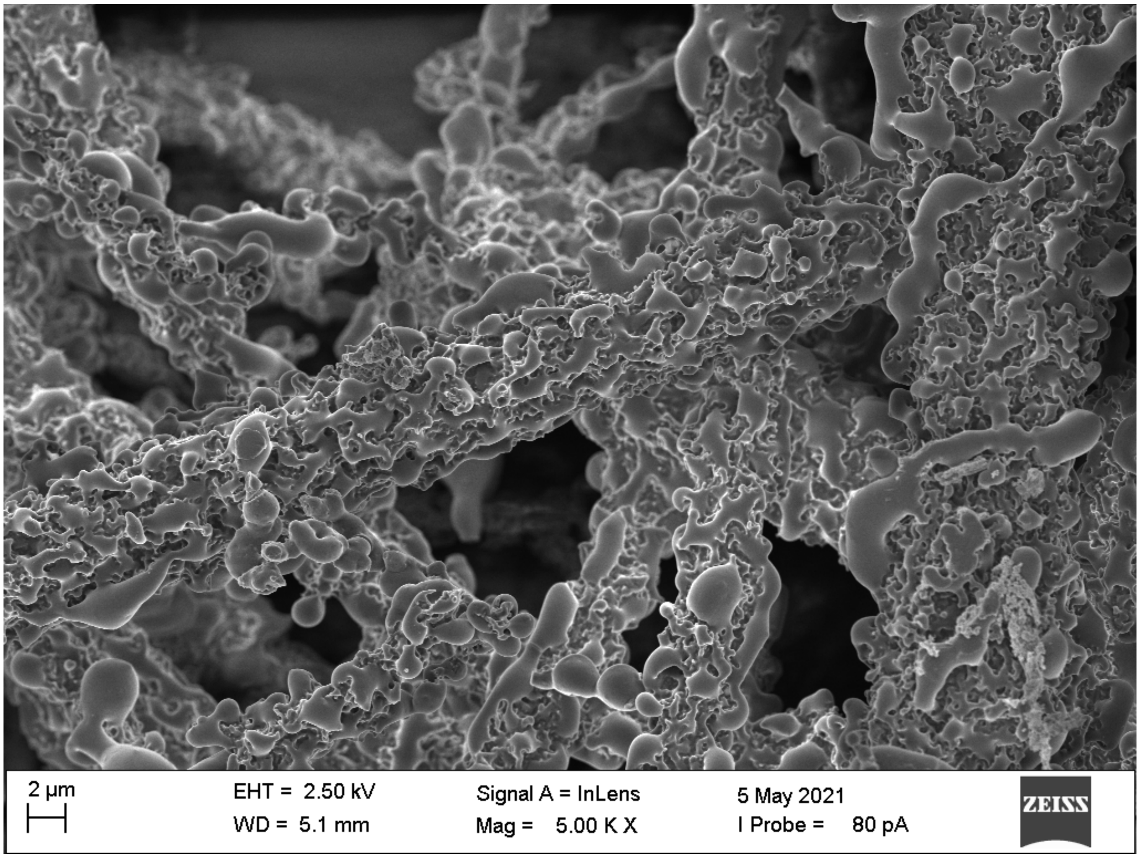

Supplementary Figure 5: SEM image of the middle (filter) layer after 4 h of irradiation.

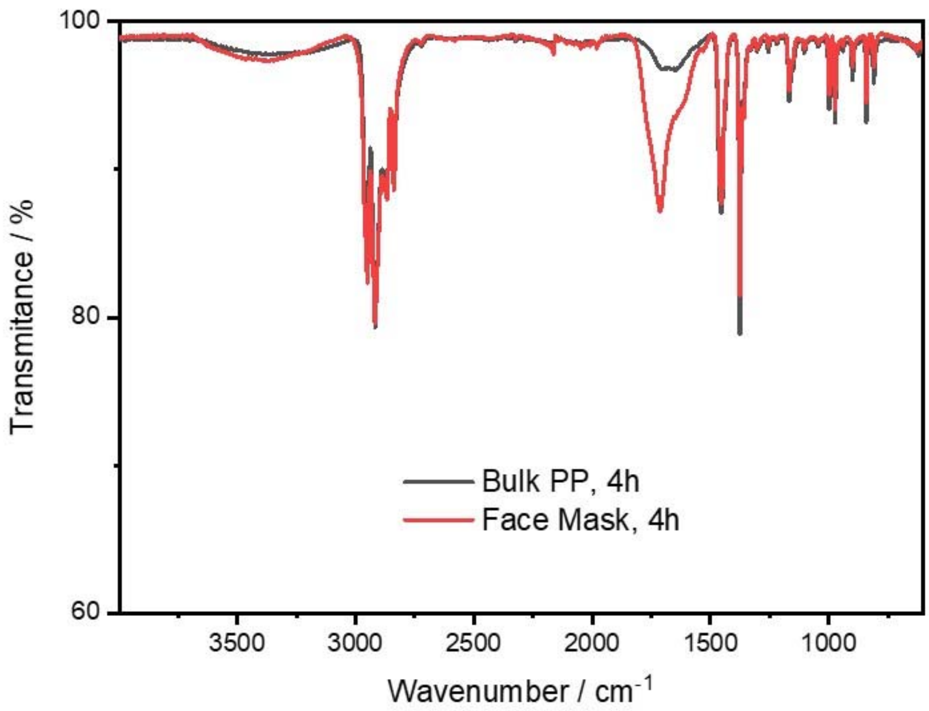

Supplementary Figure 6: ATR-IR spectra of the bulk PP after 4h of irradiation at 200 W compared to the face mask material after a comparable irradiation.

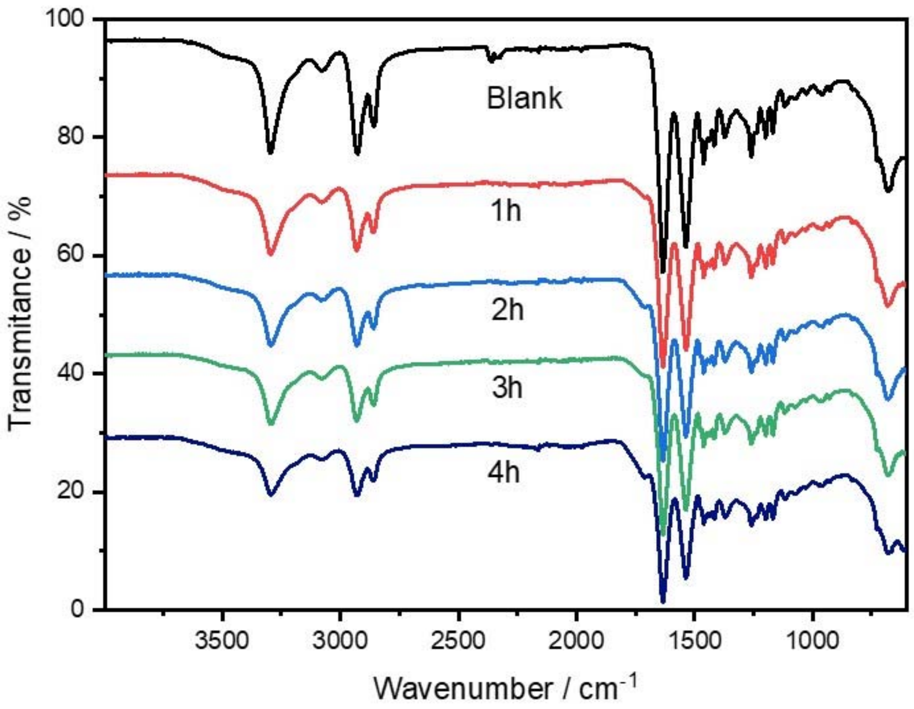

Supplementary Figure 7: ATR-IR spectra of the elasticated ear loops for un-irradiated sample and 1 h, 2 h, 3 h and 4 h irradiated samples.

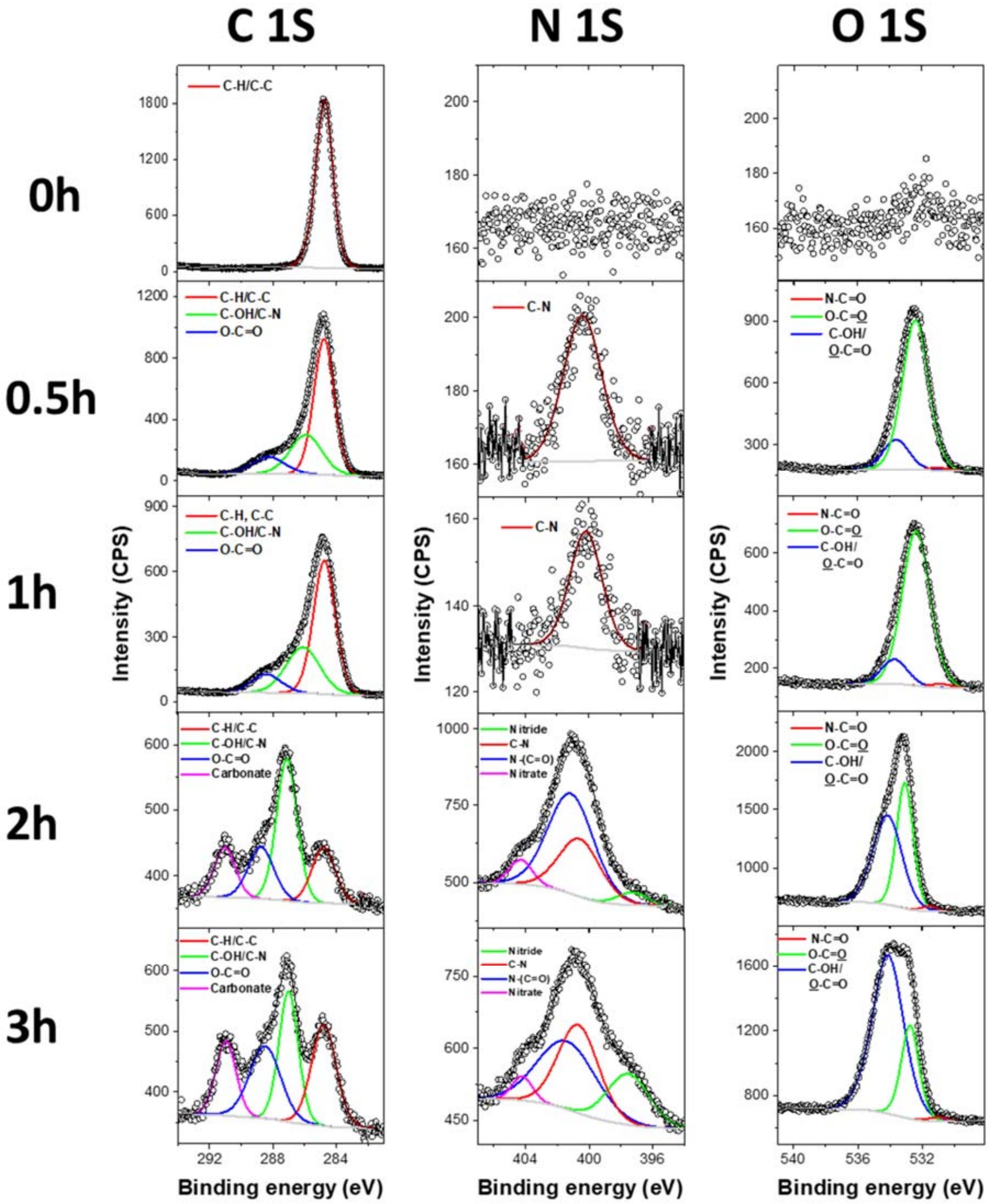

Supplementary Figure 8: XPS data from the middle (filter) layer for un-irradiated (0h) and 0.5 h, 1 h, 2 h and 3 h irradiated samples for C 1s, N 1s and O 1s.

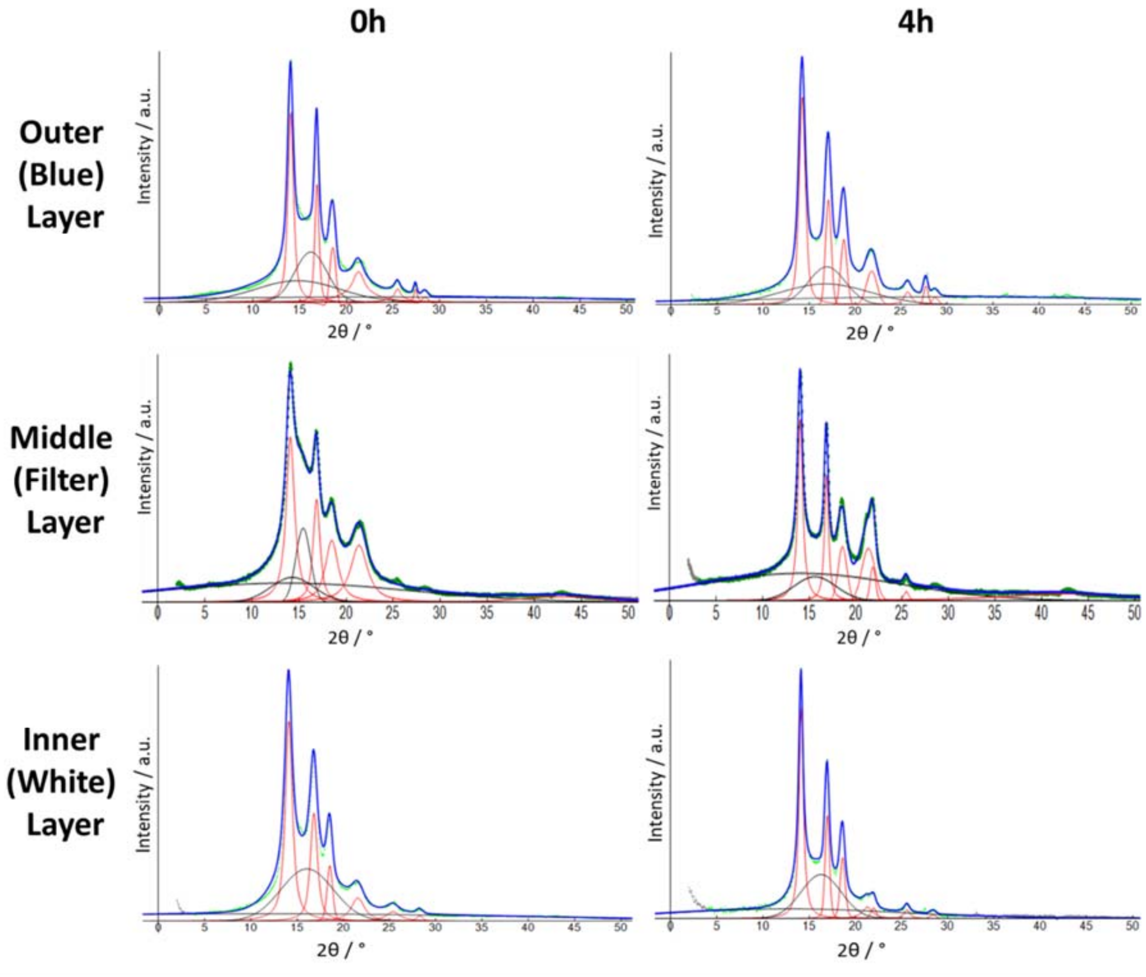

Supplementary Figure 9: Fitting of WAXS spectra for the un-irradiated and 4 h irradiated samples of the outer (blue) layer, middle (filter) layer and inner (white) layer.

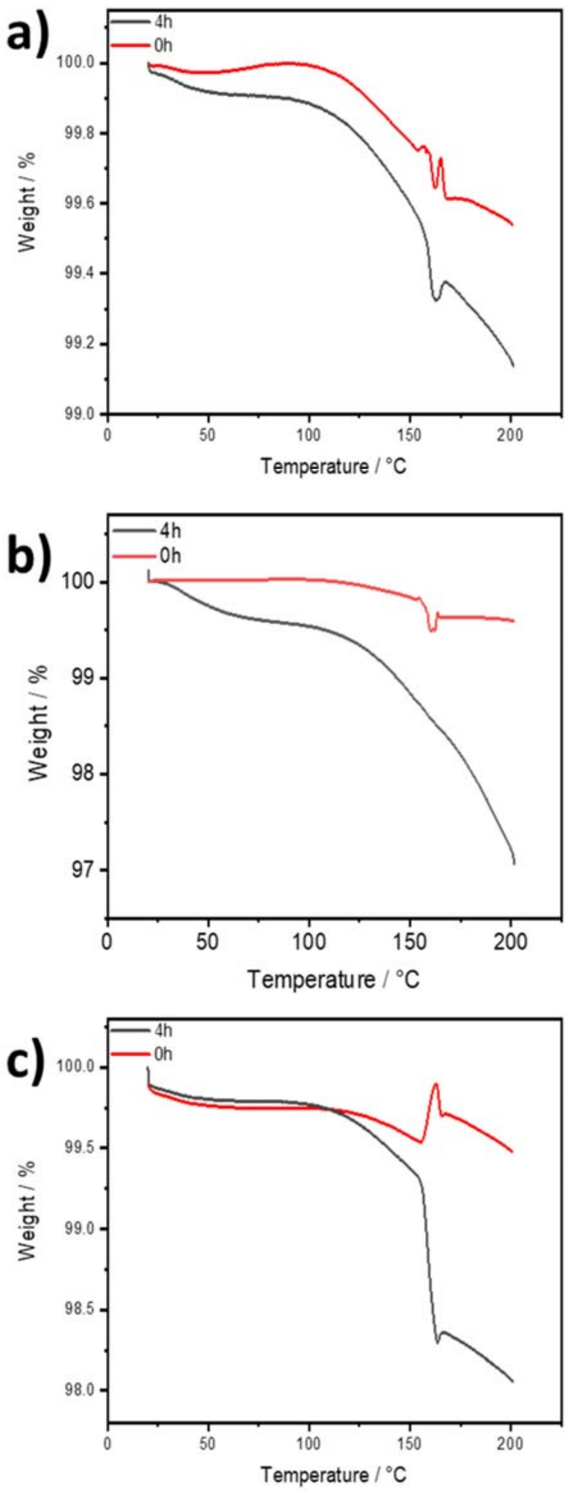

Supplementary Figure 10: TGA measurements of un-irradiated and 4 h irradiated samples of the a) outer (blue) layer, b) middle (filter) layer and c) inner (white) layer.

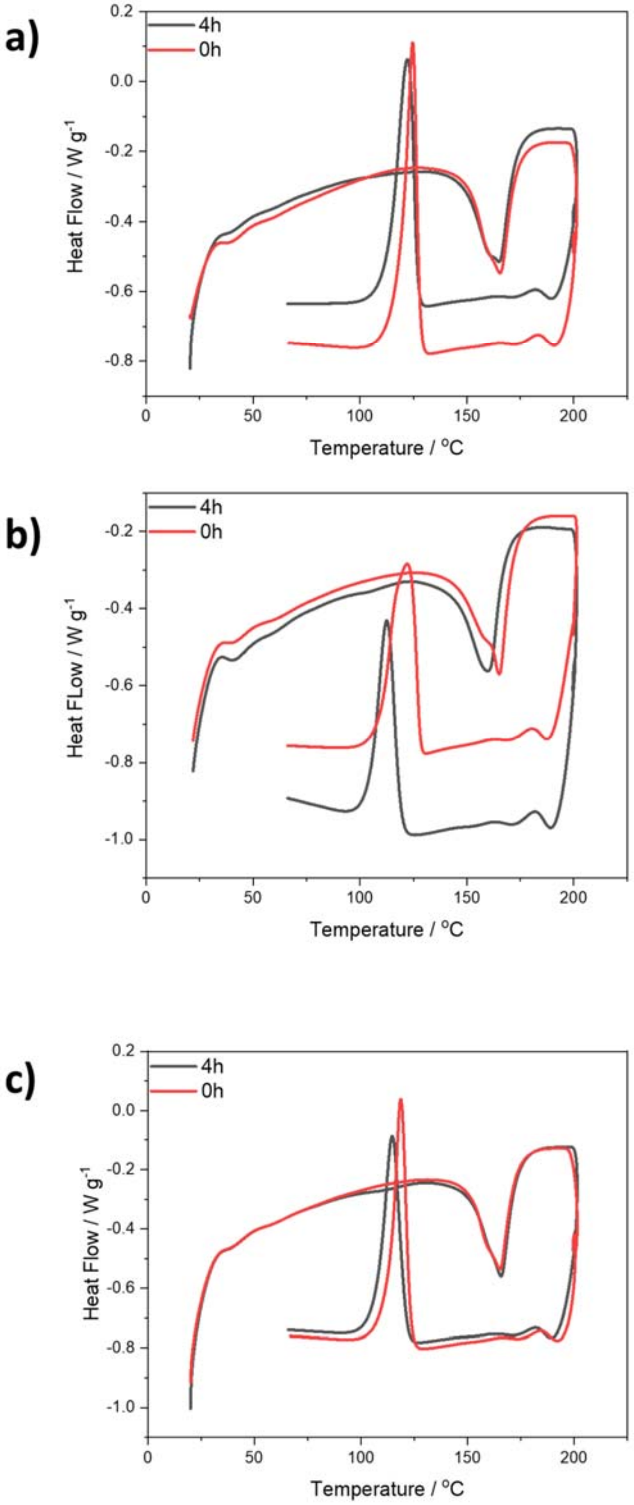

Supplementary Figure 11: DSC measurements of un-irradiated and 4 h irradiated samples of the a) outer (blue) layer, b) middle (filter) layer and c) inner (white) layer.

Sample	WAXS<br>%<br>Crystallinity	DSC<br>%<br>Crystallinity
Outer (blue) layer 0 h	34.7	42.1
Outer (blue) layer 4 h	33.4	39.7
Middle (filter) layer 0 h	47.9	42.5
Middle (filter) layer 4 h	41.9	41.8
Inner (white) layer 0 h	39.4	43.2
Inner (white) layer 4 h	39.0	41.5

Supplementary Table 1: Table showing the comparison of the percentage of crystallinity obtained by two different methods (WAXS and DSC) for un-irradiated and 4 h irradiated samples of each layer.
